# The Reasons Why Physicians Succeed

**Published:** 1907-09

**Authors:** 


### THE REASONS WHY PHYSICIANS SUCCEED.

We are often struck by the wonderful success of some individual in the practice of medicine. Do we as often stop to analyze the situation and endeavor to find out what produces such a condition in his particular case? We hear someone say that his neighbor whom he personally knows to be limited in capacity, in information and in preparation, enjoys a large part of the work of his profession in that special community. There must be a reason for this, and perhaps it is as frequently as anything else the personal equation that enters largely into the matter. There is a decision, a firmness, a kindness and an attentiveness, interest that other men do not show in their work· We think it is the rule rather than the exception that a patient would rather have a doctor who is personally interested in him, who knew comparatively little, than one who had the reputation of knowing it all and regarded the patient as he would a piece of furniture. If we are not mistaken, the time for planting this interest in each individual case dates back to the time when men are doing their hospital and clinical work. They should pay attention to every detail and every requirement of any case that is of sufficient importance to come under their observation and care. They should learn personally to be able to do any and everything that could contribute to the comfort as well as to the recovery of the individual case, for the simple reason that they cannot teach others to do that which they have not themselves already learned. It is positively painful to see bright, well educated, well informed physicians who have obtained sufficient information to secure good hospital appointments drift hopelessly and aimlessly through their service, apparently having in view only the acquisition of a hospital diploma. Indeed, it is almost certain that we can predicate what will be the future of those whom we have seen in the performance of their hospital and dispensary work. What is true of the younger members of the profession is still more true after some years of practice. If they are slipshod, careless, indifferent, inattentive, they cannot expect to hold, much less to engage a large clientele, and if some of our brethren who take every opportunity to proclaim their fitness for the practice of their profession and their inability to get sufficient work to keep their body and soul together would practice a little introspection and a litle more reformation, if not too late, they would find condition changing. In medicine as in everything else the survival of the fittest is apt to be the rule.—Ed. The Post Graduate Aug. 1907.

